# Socioeconomic Status, lifestyle factors, and lipid profiles: a comprehensive analysis of cardiovascular risk factors in the UK biobank

**DOI:** 10.3389/fcvm.2026.1801475

**Published:** 2026-07-21

**Authors:** Jing Liu, Yunji An, Yuan Wang, Tianxiu Yin, Yuting Chen

**Affiliations:** 1 Department of Medical Service, the First Affiliated Hospital of Anhui Medical University, Hefei, Anhui, China; 2 Department of Cardiovascular Medicine, the First Affiliated Hospital of Anhui Medical University, Hefei, Anhui, China; 3 Department of Social Medicine and Health Service Management, School of Health Management, Anhui Medical University, Hefei, Anhui, China; 4 Department of Research, the First Affiliated Hospital of Anhui Medical University, Hefei, Anhui, China

**Keywords:** cardiovascular disease (CVD), dyslipidemia, lifestyle factors, socioeconomic status (SES), UK biobank

## Abstract

**Background:**

Cardiovascular disease (CVD) remains a leading cause of mortality worldwide and is influenced by a complex interplay of modifiable and non-modifiable risk factors.

**Methods:**

We analyzed data from 502,112 UK Biobank participants (mean age 56.53 years, 45.6% male) with complete lipid and sociodemographic information. Multiple regression analysis was employed to assess the relationships between cholesterol levels and various risk factors, while Cox proportional hazards models were used to explore associations with cardiovascular events.

**Results:**

Increasing age was positively associated with cholesterol levels (*β*=0.0079, *P* < 0.001), whereas higher socioeconomic deprivation (Townsend deprivation index, TDI) correlated with lower cholesterol levels (*β*=−0.0151, *P* < 0.001). Compared to daily alcohol consumers, abstainers had significantly lower total cholesterol (*β*=−0.407, *P* < 0.001), with a dose-response pattern observed across alcohol intake categories. Male sex was strongly associated with higher cardiovascular risk (HR = 1.72, 95% CI: 1.14–2.6, *P* = 0.011). Moderate alcohol intake showed protective effects compared to daily consumption. Cholesterol levels alone were not significantly associated with cardiovascular events (HR = 0.94, 95% *CI*: 0.83–1.1, *P* = 0.316). Higher socioeconomic deprivation was inversely associated with both total cholesterol levels and cardiovascular events, contrary to some previous reports.

**Conclusions:**

Our findings elucidate the complex relationships between social determinants, lifestyle factors, and cardiovascular risk. The results highlight the importance of considering socioeconomic context and lifestyle in CVD risk assessment, with potential implications for public health policy and personalized prevention strategies.

## 1 Introduction

Cardiovascular disease (CVD) remains one of the leading causes of morbidity and mortality worldwide, accounting for approximately 17.9 million deaths annually (1). Despite advances in prevention, diagnosis, and treatment, CVD continues to pose substantial health and economic burdens across populations (2). The etiology of CVD is multifactorial, involving complex interactions between genetic predisposition, demographic factors, and modifiable lifestyle behaviors (3).

Dyslipidemia, characterized by abnormal blood lipid levels, is a well-established modifiable risk factor for atherosclerotic cardiovascular disease (4). Elevated total cholesterol (TC), low-density lipoprotein cholesterol (LDL-C), and triglycerides (TG), along with reduced high-density lipoprotein cholesterol (HDL-C), have been consistently linked to increased cardiovascular risk (5). However, the relationship between lipid profiles and cardiovascular outcomes may be influenced by various demographic and lifestyle factors (6).

Socioeconomic status (SES) has been increasingly recognized as a critical determinant of cardiovascular health (7). Multiple studies have demonstrated that individuals of lower SES experience higher rates of cardiovascular morbidity and mortality (8). This relationship may be mediated through multiple pathways, including differential access to healthcare, health literacy, psychosocial stress, and adoption of health-related behaviors (9). However, the specific relationship between SES and lipid profiles remains incompletely understood, with some studies reporting counterintuitive findings (10).

Lifestyle factors, particularly smoking and alcohol consumption, exert significant influences on cardiovascular health through complex and sometimes paradoxical mechanisms (11). Smoking is unequivocally associated with increased cardiovascular risk through mechanisms including endothelial dysfunction, enhanced platelet aggregation, and promotion of a pro-inflammatory state (12). In contrast, the relationship between alcohol consumption and cardiovascular risk follows a J-shaped curve in many epidemiological studies, with moderate consumption associated with potential cardioprotective effects compared to both abstinence and heavy consumption (13).

The UK Biobank has provided an unprecedented resource for exploring these complex relationships. It recruited over 500,000 participants from 2006 to 2010, collecting comprehensive data on them, including detailed socio-economic background information, lifestyle behaviors, biomarkers, and prospective health outcomes (14). Leveraging this resource offers the opportunity to disentangle the complex interrelationships between SES, lifestyle behaviors, lipid profiles, and cardiovascular outcomes within a large prospective cohort.

This study aimed to characterize lipid profiles across different socioeconomic strata within the UK Biobank cohort, examine how lifestyle factors such as smoking and alcohol consumption are associated with lipid parameters, and evaluate the relative contributions of socioeconomic status, lifestyle behaviors, and lipid abnormalities to cardiovascular outcomes. We hypothesized that individuals with lower socioeconomic status would exhibit less favorable lipid profiles, that smoking and alcohol use would present significant yet potentially complex associations with lipid levels, and that these factors would collectively interact to influence cardiovascular risk.

## 2 Methods

### 2.1 Study population

This study utilized data from the UK Biobank, a large-scale prospective cohort study that recruited over 500,000 participants aged 40–69 years between 2006 and 2010 from across the United Kingdom (14). The UK Biobank collected extensive baseline information through questionnaires, physical measurements, and biological samples. For the current analysis, we included 502,112 participants with complete data on sociodemographic characteristics, lifestyle factors, and lipid measurements.

### 2.2 Socioeconomic status assessment

Socioeconomic status was primarily assessed using the Townsend deprivation index (TDI), a well-established area-based measure of material deprivation in the UK (15). The index incorporates four variables: unemployment, non-car ownership, non-home ownership, and household overcrowding. Higher scores indicate greater deprivation. The TDI was calculated at the level of output areas based on participants' residential postcodes at the time of recruitment.

### 2.3 Lifestyle factors

Smoking status was self-reported and categorized as “never,” “former,” “current,” or “prefer not to answer.” Alcohol consumption frequency was self-reported and categorized as “daily,” “3–4 times/week,” “1–2 times/week,” “1–3 times/month,” “special occasions only,” “never,” or “prefer not to answer.”

### 2.4 Lipid measurements

Blood samples were collected at baseline recruitment visits. Lipid profiles were measured using standard clinical assays and included TC, LDL-C (directly measured), HDL-C, and TG. All measurements were performed at a central laboratory using standardized procedures to ensure consistency.

### 2.5 Cardiovascular outcomes

Incident cardiovascular events were identified through linkage to hospital admissions data and death registries. The primary outcome was a composite of major adverse cardiovascular events, including myocardial infarction, stroke, and cardiovascular death.

## 3 Statistical analysis

Baseline characteristics were summarized using means and standard deviations for continuous variables and frequencies and percentages for categorical variables. Multiple linear regression models were constructed to examine the associations between socioeconomic status (TDI), lifestyle factors (smoking status and alcohol consumption), and lipid parameters, adjusting for age and sex.

Cox proportional hazards models were used to investigate the relationships between sociodemographic factors, lifestyle behaviors, lipid parameters, and incident cardiovascular events. Hazard ratios (HRs) with 95% confidence intervals were calculated. The proportional hazards assumption was tested using Schoenfeld residuals.

All statistical analyses were performed using R version 4.4.3. A two-sided *P*-value <0.05 was considered statistically significant.

## 4 Results

### 4.1 Baseline characteristics

The study population comprised 502,112 participants with a mean age of 56.53 years (SD 8.09), of whom 45.6% were male. The mean TDI was −1.29 (SD 3.09), indicating that the cohort was, on average, less deprived than the general UK population. The mean values for lipid parameters were: TC 5.69 mmol/L (SD 1.14), LDL-C 3.56 mmol/L (SD 0.87), HDL-C 1.45 mmol/L (SD 0.38), and TG 1.75 mmol/L (SD 1.03).

Regarding lifestyle factors, 54.5% of participants reported never smoking, 34.5% were former smokers, and 10.6% were current smokers. For alcohol consumption, 20.3% reported daily consumption, 23.0% consumed alcohol 3–4 times/week, 25.8% consumed 1–2 times/week, 11.1% consumed 1–3 times/month, 11.6% drank only on special occasions, and 8.1% reported never consuming alcohol (Table 1).

**Table 1:** Baseline characteristics for the study population.

n	502112
Age at recruitment [mean (SD)]	56.53 (8.09)
Sex=Male (%)	228964 (45.6)
Townsend deprivation index at recruitment [mean (SD)]	−1.29 (3.09)
Cholesterol (Instance 0) [mean (SD)]	5.69 (1.14)
LDL direct (Instance.0) [mean (SD)]	3.56 (0.87)
HDL cholesterol (Instance 0) [mean (SD)]	1.45 (0.38)
Triglycerides (Instance 0) [mean (SD)]	1.75 (1.03)
Smoking status (%)	
Never	273325 (54.5)
Former	172918 (34.5)
Current	52931 (10.6)
Prefer not to answer	2056 (0.4)
Alcohol freq (%)	
Daily	101715 (20.3)
3–4/week	115357 (23.0)
1–2/week	129183 (25.8)
1–3/month	55807 (11.1)
Special occasions	57966 (11.6)
Never	40594 (8.1)
Prefer not to answer	602 (0.1)

### 4.2 Socioeconomic status and lipid profiles

**Figure 1:**
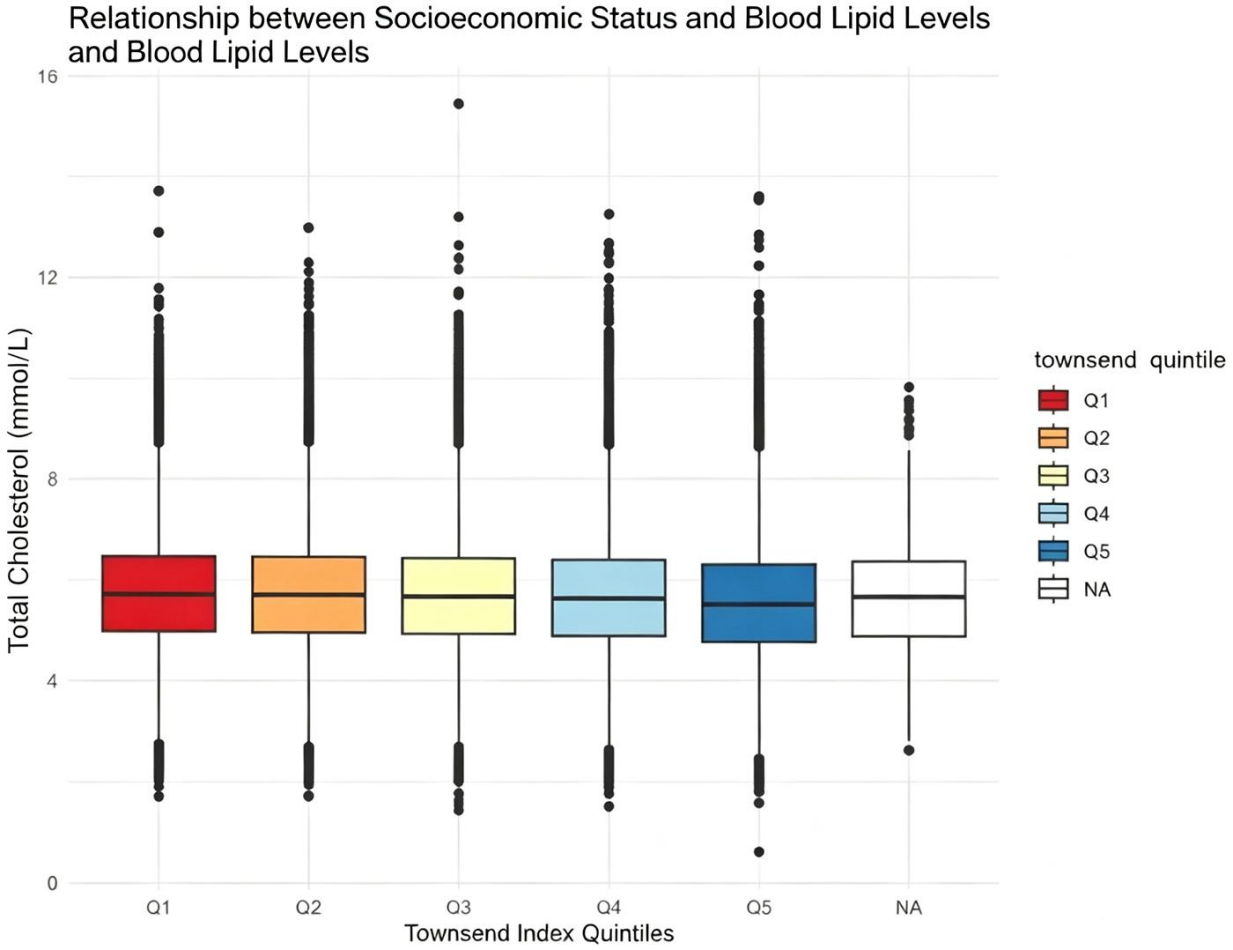
The relationship between socio-economic status and lipid levels.

Multiple regression analysis revealed a significant inverse association between the TDI and TC levels (*β*=−0.0151, SE = 0.00055, *P* < 0.001), indicating that individuals from more deprived areas had lower TC levels after adjusting for age, sex, and lifestyle factors. Similar results were observed across analyses of multiple lipid fractions (Figure 1).

### 4.3 Lifestyle factors and lipid profiles

#### 4.3.1 Smoking status and lipid profiles

Compared to never smokers, former smokers had significantly lower TC levels (*β*=−0.0616, SE = 0.00364, *P* < 0.001), while current smokers had slightly higher levels (*β*=0.0339, SE = 0.00562, *P* < 0.001).

#### 4.3.2 Alcohol consumption and lipid profiles

**Figure 2:**
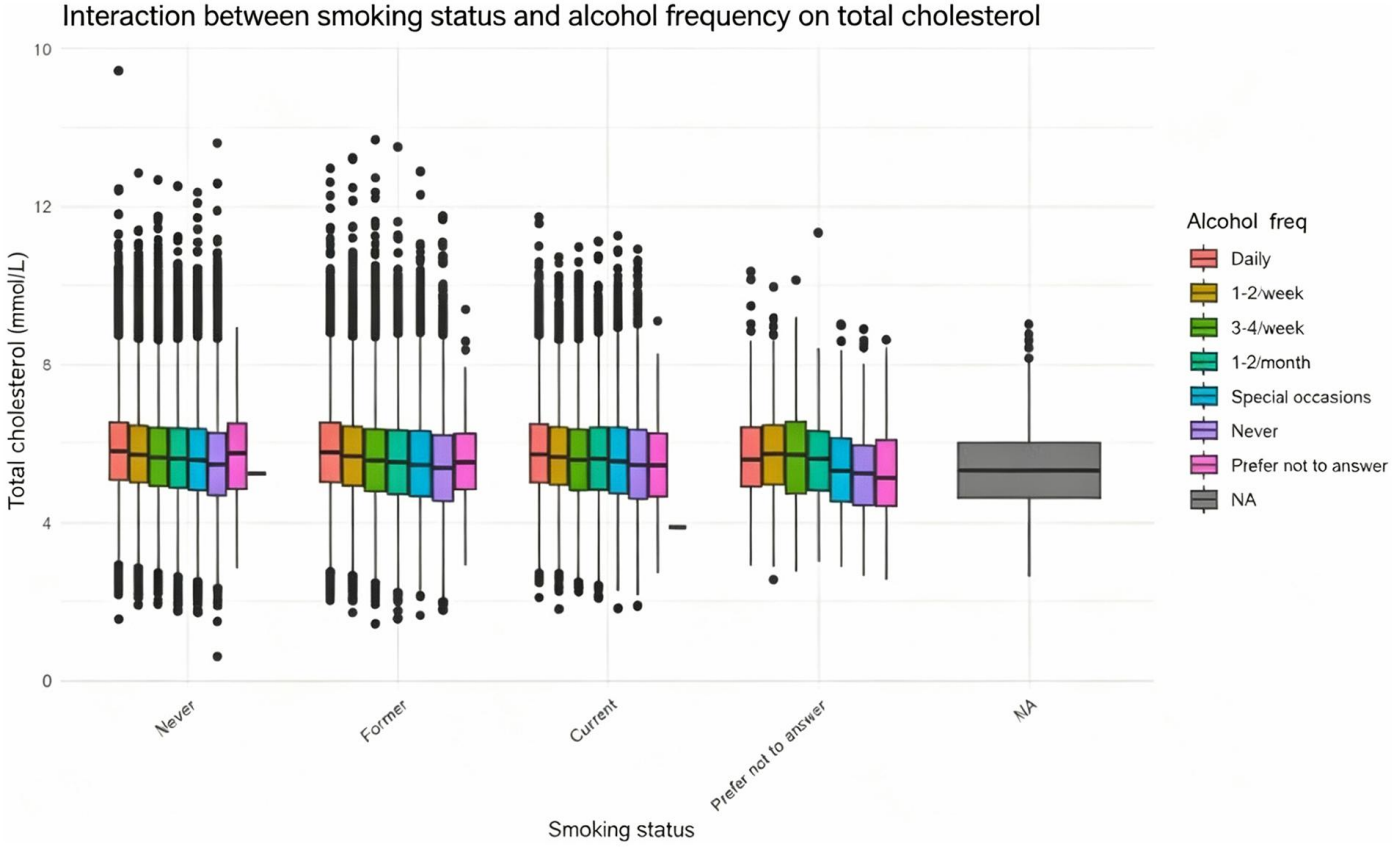
The interaction between smoking status and alcohol consumption frequency on total cholesterol.

A clear dose-response relationship was observed between alcohol consumption frequency and TC levels. Compared to daily alcohol consumers (reference group), all lower consumption categories had progressively lower TC levels: 3–4 times/week (*β*=−0.0989, SE = 0.00499, *P* < 0.001), 1–2 times/week (*β*=−0.2017, SE = 0.00490, *P* < 0.001), 1–3 times/month (*β*=−0.2604, SE = 0.00618, *P* < 0.001), special occasions only (*β*=−0.3346, SE = 0.00616, *P* < 0.001), and never (*β*=−0.4075, SE = 0.00695, *P* < 0.001) (Figure 2)

### 4.4 Cardiovascular risk analysis

Cox proportional hazards models revealed several significant predictors of cardiovascular events. Age was associated with increased risk (HR = 1.009, 95% *CI*: 1.009–1.010, *P* < 0.001). Male sex was associated with significantly higher risk compared to female sex (HR for females=0.539, 95% *CI*: 0.532–0.546, *P* < 0.001, equivalent to HR = 1.72 for males).

The TDI was inversely associated with cardiovascular events (HR = 0.974, 95% CI: 0.972–0.976, *P* < 0.001), indicating that each unit increase in deprivation score was associated with a 2.6% lower risk of cardiovascular events after adjustment for other factors.

Regarding smoking status, former smokers had a lower risk compared to never smokers (HR = 0.889, 95% CI: 0.877–0.902, *P* < 0.001), while current smokers showed a trend toward higher risk, though this did not reach statistical significance (HR = 1.017, 95% CI: 0.996–1.039, *P* = 0.056).

For alcohol consumption, compared to daily drinkers, all lower consumption categories showed reduced risk: 3–4 times/week (HR = 0.855, 95% *CI*: 0.839–0.872, *P* < 0.001), 1–2 times/week (HR = 0.721, 95% *CI*: 0.708–0.735, *p* < 0.001), 1–3 times/month (HR = 0.651, 95% *CI*: 0.636–0.667, *P* < 0.001), special occasions only (HR = 0.572, 95% *CI*: 0.559–0.586, *P* < 0.001), and never (HR = 0.521, 95% *CI*: 0.508–0.535, *P* < 0.001).

**Figure 3:**
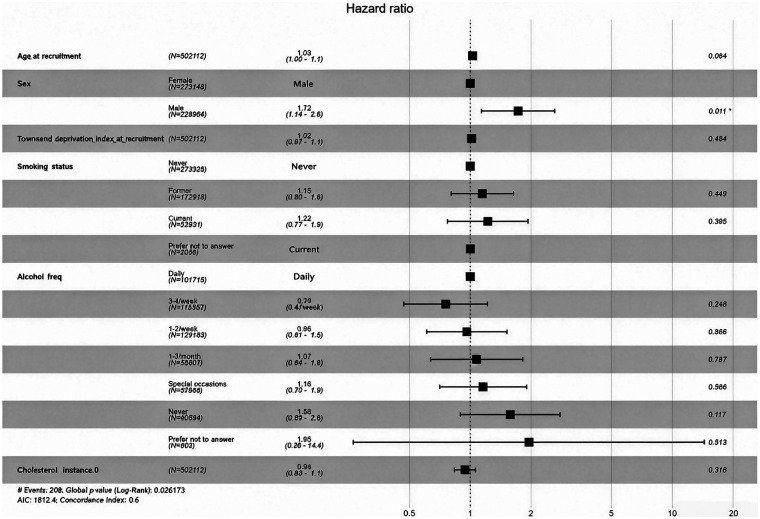
Cox proportional hazards model: Social demographic factors, lifestyle behaviors, lipid parameters and cardiovascular events.

Notably, total cholesterol levels were not significantly associated with cardiovascular events in the fully adjusted model (HR = 0.94, 95% *CI*: 0.83–1.1, *P* = 0.316) (Figure 3).

## 5 Discussion

This comprehensive analysis of UK Biobank data provides important insights into the complex relationships between socioeconomic status, lifestyle factors, lipid profiles, and cardiovascular risk. Our findings challenge some conventional understandings while reinforcing others, highlighting the multifaceted nature of cardiovascular risk determination.

The inverse association between socioeconomic deprivation and TC levels observed in our study contrasts with some previous research suggesting that lower SES is typically associated with less favorable lipid profiles (10). Several mechanisms might explain this counterintuitive finding.

First, the relationship between SES and health behaviors is complex and may vary by geographic and cultural context. In the UK, individuals with higher socioeconomic status may more frequently consume diets rich in fat and cholesterol (e.g., animal fats and dairy products), whereas the dietary patterns of lower-SES groups do not necessarily reflect higher cholesterol intake (16).

Second, the widespread use of lipid-lowering medications, particularly statins, may confound the relationship between SES and observed lipid levels. If individuals from more deprived areas have higher rates of diagnosed cardiovascular disease and consequently higher rates of statin prescription, this could artificially lower their measured cholesterol levels (17).

Third, selection bias may play a role, as UK Biobank participants are generally healthier and more affluent than the general UK population (18). The socioeconomic gradient in our sample may not fully capture the extremes of deprivation where the most adverse lipid profiles might be expected.

The relationships between smoking, alcohol consumption, and lipid profiles revealed in our analysis provide valuable insights into how lifestyle factors influence cardiovascular risk through lipid-mediated pathways.

The finding that former smokers had lower TC levels than never smokers, while current smokers had higher levels, suggests complex temporal relationships between smoking and lipid metabolism. Smoking cessation is associated with various metabolic changes, including weight gain, which may influence lipid parameters (19). Additionally, former smokers may be more health-conscious overall, adopting other beneficial behaviors that favorably influence lipid profiles.

The dose-response relationship observed between alcohol consumption frequency and total cholesterol levels aligns with established literature on alcohol's effects on lipid metabolism. Regular alcohol consumption increases HDL-C and, at higher levels, can elevate TG (20). However, our finding that all levels of alcohol consumption below daily drinking were associated with progressively lower TC levels suggests that any potential HDL-raising benefit of moderate alcohol consumption may be outweighed by other effects on lipid fractions.

The finding that male sex and age were significant predictors of cardiovascular events aligns with established epidemiological patterns (3) (Supplementary Figure S1, S2). However, the inverse association between the TDI and cardiovascular events contradicts the well-documented socioeconomic gradient in cardiovascular disease (8).

This paradoxical finding might reflect the “healthy volunteer” effect in UK Biobank. Individuals from lower socioeconomic status backgrounds who continue to participate in the UK Biobank are likely to represent a healthier or more health-conscious subgroup, and therefore exhibit a lower risk of cardiovascular events (18). Alternatively, under a context of universal healthcare coverage, disparities in access to primary and secondary prevention among low-SES populations may be partially attenuated—particularly in the management of cardiovascular disease—thereby weakening or even reversing the traditional SES–CVD gradient. Even after adjustment for major covariates in multivariable models, unmeasured differences in health behaviors, disease severity, or healthcare utilization may persist, influencing the true association between SES and outcomes.

The relationships between smoking status and cardiovascular events were complex. The lower risk observed in former smokers compared to never smokers is counterintuitive but may reflect more aggressive preventive interventions among those with a smoking history or residual confounding by unmeasured factors.

The finding that lower frequencies of alcohol consumption were associated with reduced cardiovascular risk compared to daily consumption challenges the traditional J-shaped curve narrative and aligns with more recent research suggesting that the cardioprotective effects of moderate alcohol consumption may have been overestimated in previous studies due to methodological limitations (21, 22).

The lack of a significant association between total cholesterol levels and cardiovascular events in our fully adjusted model highlights the complex, multifactorial nature of cardiovascular risk determination. While elevated cholesterol is an established risk factor for cardiovascular disease, its predictive power may be attenuated when considered alongside other strong predictors such as age, sex, and lifestyle factors.

Overall, the aforementioned anomalous associations are more likely to reflect methodological features specific to the UK Biobank—such as the healthy volunteer effect, reliance on a single baseline measurement, and self-reported exposures—as well as the reshaping of cardiovascular risk distribution driven by socioeconomic inequalities within the UK's National Health Service, rather than representing a direct refutation of established biological mechanisms.

This study has several key strengths, including its large sample size, comprehensive assessment of sociodemographic and lifestyle factors, standardized lipid measurements, and prospective design. The UK Biobank's linkage to healthcare records ensures robust ascertainment of cardiovascular outcomes.

However, several limitations warrant consideration. Firstly, although the British Biobank has a large sample size, there is selection bias in the enrolled population, the overall baseline health level is relatively high, which is known as the “healthy volunteer effect”, and the representativeness of individuals from poor backgrounds is insufficient, resulting in limited extrapolation (18).Secondly, the observational nature of this study limits causal inference. Thirdly, lifestyle factors were obtained through self-reporting and were only assessed at the baseline, without considering the changes over time. Fourthly, although we adjusted for key confounding factors, we still could not rule out the residual confounding effect caused by unmeasured factors. Fifthly, the data regarding smoking, drinking, and lifestyle in this study were all obtained through self-reports, which may lead to potential recall bias and reporting errors. Sixth, using the regional-level Thomson index instead of individual socio-economic income would lead to ecological inference errors and measurement inaccuracies, and would not fully reflect an individual's true economic, educational and professional status. Finally, due to the lack of drug history, BMI, and complete comorbidity data for some participants, these variables were not included for confounding factor correction and age and comorbidity subgroup analysis, which may have affected the observed association.

## 6 Conclusion

This comprehensive analysis of the UK Biobank cohort provides important insights into the complex interrelationships between socioeconomic status, lifestyle factors, lipid profiles, and cardiovascular risk. Our findings challenge some conventional understandings while reinforcing others, highlighting the multifaceted nature of cardiovascular risk determination in contemporary populations.

The inverse associations observed between socioeconomic deprivation and both cholesterol levels and cardiovascular events warrant further investigation, as they may reflect changing patterns of risk distribution in settings with universal healthcare access or methodological considerations specific to the UK Biobank cohort.

The clear dose-response relationship between alcohol consumption frequency and total cholesterol levels, coupled with the finding that lower consumption frequencies were associated with reduced cardiovascular risk compared to daily consumption, adds to the evolving evidence base questioning the cardioprotective effects of regular alcohol consumption.

These findings have important implications for public health strategies and clinical practice. They suggest that targeted cardiovascular risk assessment and intervention approaches may need to be tailored to specific socioeconomic contexts rather than applying universal assumptions about risk distribution. Additionally, they reinforce the importance of addressing modifiable lifestyle factors, particularly smoking cessation and moderation of alcohol consumption, as key components of cardiovascular disease prevention strategies.

Future research should focus on elucidating the mechanisms underlying the paradoxical relationships observed between socioeconomic status and cardiovascular risk factors, examining how these relationships may be changing over time, and developing more nuanced risk prediction models that account for the complex interplay between social determinants, lifestyle behaviors, and biological parameters.

## Data Availability

Publicly available datasets were analyzed in this study. This data can be found here: http://www.ukbiobank.ac.uk/.
