# Supplementary material for: Socioeconomic Status, lifestyle factors, and lipid profiles: a comprehensive analysis of cardiovascular risk factors in the UK biobank
Source: Front Cardiovasc Med. 2026 Jul 21;13:1801475. doi: 10.3389/fcvm.2026.1801475 (PMC13433360; doi:10.3389/fcvm.2026.1801475)
Supplement: Supplementary file 1 [file Datasheet1.docx]

**Supplementary Information**

**Socioeconomic Status, Lifestyle Factors, and Lipid Profiles: A Comprehensive Analysis of Cardiovascular Risk Factors in the UK Biobank**

**Supplementary Fig.1.**Trend of Cholesterol Levels with Age

**Supplementary Fig.2.**Total Cholesterol Levels by Age and Gender
